# Response to commentaries

**DOI:** 10.1111/add.12890

**Published:** 2015-05-11

**Authors:** Marina Davoli, Laura Amato, David Tovey

**Affiliations:** ^1^ Department of EpidemiologyLazio Regional Health ServiceRomeItaly; ^2^ Editor‐in‐Chief, Cochrane Editorial UnitCochrane Central ExecutiveLondonUK

**Keywords:** Benzodiazepines, children, motherhood, pregnancy, register studies, risk families

The authors of the two commentaries 1, 2 raise interesting issues about the role of Cochrane Systematic Reviews in informing international guidelines, and give us the opportunity to describe some of the ongoing efforts of Cochrane to address these challenges. For this reason, we invited Cochrane's Editor‐in‐Chief to join us in preparing our response.

The first challenge is the mismatch between what Cochrane can offer and what the World Health Organization (WHO) actually needs; the author underlined that: ‘for some questions, and for several outcomes that are key for guideline developers, no data are available from Cochrane reviews’. This is indeed true, but whether a Cochrane Review can draw useful conclusions depends upon results from primary studies being available and sufficient. One of the most frequent mismatches between the wishes of guidelines providers and what can be produced in Cochrane Reviews relates to the breadth of the PICO (Population, Intervention, Comparison, Outcomes) elements covered. It is frequently the case that guideline producers require greater breadth (e.g. multiple subgroups) than the evidence can cover without threatening its validity.

The lack of well‐conducted primary studies addressing relevant questions and outcomes is therefore a concern, therefore every Cochrane Review includes an ‘implication for research’ section which focuses on future research needs, in terms of outcomes and participants, but also setting priorities and identifying areas of uncertainty.

We acknowledge that an issue of prioritization also exists for Cochrane Reviews themselves. A major effort has been made in recent years to ensure that Cochrane Reviews address the questions and uncertainties of most importance to decision‐makers. Cochrane has also developed a partnership with the Guidelines International Network. Through this, we seek to work actively with guidelines producers to ensure that Cochrane Reviews meet the producers’ needs to the greatest extent possible.

The second challenge relates to the fact that most Cochrane Reviews include only randomized trials, although there are Cochrane groups that have always included non‐randomized studies routinely. We recognize that for some outcomes, in particular those that are rare or delayed in onset, or both, the opportunity of evaluating the evidence from non‐randomized studies is crucial to guide decisions. This raises additional challenges, including those of retrieving relevant studies, and evaluating the risk and direction of bias in such studies. Cochrane contributors, including the leadership of the Cochrane Drugs and Alcohol Group, have recently been engaged in an important project to develop a risk of bias tool for non‐randomized studies.

The third challenge relates to the comprehensiveness of the search; Barbui states that: ‘if Cochrane reviews systematically miss a proportion of evidence from low or middle income countries (LMICs), then their relevance in informing WHO recommendations, which are especially focused on the needs of LMICs, cannot be expected to be very high’. All Cochrane editorial teams include information specialists who develop expertise in locating and retrieving reports from high‐quality studies in their discipline, and many of these hand‐search relevant journals to identify such studies. However, we are not complacent. We would be pleased to benefit from the expertise of those who are familiar with the literature based in LMICs, and welcome a collaborative approach.

The fourth concern, raised by Ferri, relates to the need of ‘negotiation among panel members in order to reach a final agreement on the recommendations’. We agree that health‐care decision‐making is complex. The GRADE working group has put a major effort into making this process more explicit and transparent 3.

The DECIDE (Developing and Evaluating Communication strategies to support Informed Decisions and practice based on Evidence) project, a GRADE (Grading of Recommendations Assessment, Development and Evaluation) working group initiative funded by the European Union, has developed Evidence to Decision (EtD) frameworks for different types of decisions and recommendations. The purpose of EtD frameworks is to help panels to use evidence in a structured and transparent way to reach decisions about clinical recommendations, coverage and health system and public health interventions. The EtD frameworks have been developed to make explicit judgements about benefits and harms of the options, values, resource use, equity, acceptability and feasibility 4.

Ferri also states: ‘…the time allowed for decision‐making, is short compared with the time required for accurate systematic reviewing and recommendation development’. We recognize the need to ensure the efficiency of the review production process, and Cochrane is working in a number of areas to accelerate this. It is also implementing rapid review and focused updating services. Nevertheless, this issue also involves the timely and complete publication of data from primary research. No matter how rapid we can be, if we do not have available evidence to answer relevant questions, reviews based on limited data will be flawed.

Finally, concerning the complex nature of systematic review reports and the challenges of extracting key information: we recognize and appreciate that Cochrane Reviews can be lengthy and complex, and this has been one of the key drivers to implement the GRADE approach and Summary of Findings tables. While even these can seem intimidating at first, research has shown that GRADE Summary of Findings tables are effective in enabling readers to understand the results of a review accurately and to place it into the context of their experience.

## Declaration of interests

Marina Davoli and Laura Amato are respectively coordinating editor and managing editor of the Cochrane Drugs and Alcohol group and are both members of the GRADE working group. They never received any funding from alcohol, pharmaceutical or tobacco companies. David Tovey is the editor in chief of the Cochrane Collaboration.
